# Multichannel-Sensing Scheduling and Transmission-Energy Optimizing in Cognitive Radio Networks with Energy Harvesting

**DOI:** 10.3390/s16040461

**Published:** 2016-03-31

**Authors:** Tran-Nhut-Khai Hoan, Vu-Van Hiep, In-Soo Koo

**Affiliations:** The School of Electrical Engineering, University of Ulsan, Ulsan 680-749, Korea; tnkhoan@ctu.edu.vn (T.-N.-K.H.); vvhiep@gmail.com (V.-V.H.)

**Keywords:** cognitive radio, energy harvesting, throughput, multichannel sensing schedule, transmission energy optimization

## Abstract

This paper considers cognitive radio networks (CRNs) utilizing multiple time-slotted primary channels in which cognitive users (CUs) are powered by energy harvesters. The CUs are under the consideration that hardware constraints on radio devices only allow them to sense and transmit on one channel at a time. For a scenario where the arrival of harvested energy packets and the battery capacity are finite, we propose a scheme to optimize (i) the channel-sensing schedule (consisting of finding the optimal action (silent or active) and sensing order of channels) and (ii) the optimal transmission energy set corresponding to the channels in the sensing order for the operation of the CU in order to maximize the expected throughput of the CRN over multiple time slots. Frequency-switching delay, energy-switching cost, correlation in spectrum occupancy across time and frequency and errors in spectrum sensing are also considered in this work. The performance of the proposed scheme is evaluated via simulation. The simulation results show that the throughput of the proposed scheme is greatly improved, in comparison to related schemes in the literature. The collision ratio on the primary channels is also investigated.

## 1. Introduction

Recently, due to the developments in wireless communications technology, the prime objective of enhancing the data rate under limited spectrum resources has been challenging many researchers. According to a Federal Communications Commission spectrum policy task force report [1], utilization of the licensed spectrum varies between 15% and 80%. Therefore, to solve the spectrum scarcity issue, cognitive radio (CR) technology [2] was proposed as a viable solution by utilizing vacant licensed bandwidth or spectrum holes. CR technology allows an unlicensed user, or cognitive user (CU), to determine (via spectrum sensing [2,3]) the activity of the licensed user, or primary user (PU), on the licensed spectrum and then utilize any vacant spectrum band for transmission. When the PU is detected as active on a channel, the CU has to vacate that channel instantly and switch to another free channel to continue the transmission process. Therefore, efficient utilization of the spectrum is related to better spectrum sensing methods. However, in practical scenarios, constraints in the physical layer, such as imperfect sensing [4,5,6,7,8] and channel switching delay [9], directly affect the utilization of the vacant spectrum and, subsequently, the throughput of the cognitive radio networks (CRNs). The constraints in radio devices that limit CU sensing of all potential channels simultaneously [10,11] also restrict spectrum utilization and the throughput of CUs in multi-channel CRNs.

In addition, harvesting power and green energy-powered CRNs have recently gained considerable attention [12,13,14,15,16,17,18,19,20,21]. Specifically, the architecture, technical challenges, performance and applications of green energy-powered CRNs have been analyzed in [12,19,20,21] and the references therein. Although there have been many efforts to improve energy conversion efficiency, the harvested capacity has been limited [13]. However, green power is still envisioned as an important energy resource to liberate CRNs from energy constraints in the future. Meanwhile, state-of-the-art energy-efficient harvesting energy-powered CRNs have been investigated from various aspects, such as: solar energy harvesting-powered cognitive metro cellular network [16], cognitive radio transmitting-harvesting energy [16,17], relay, optimal energy allocation, transmission energy optimization [12,18,22,23,24] and packet transmission period optimization [25]. Obviously, the constraints in battery capacity and energy-harvested capacity should be considered as one of the major design criteria for future green-powered CRNS.

In CRNs, the optimal sensing order of the multi-channel has been considered in several studies [10,11,26,27] to improve the throughput of CR systems. However, all of these schemes were designed for infinite battery capacity systems without energy harvesters, so transmission power is not limited. In addition, the authors have not considered channel switching delay between channels [10,11,26,27], errors in spectrum sensing [26] and the correlation in spectrum occupancy across time and frequency [10,26,27], even although some measurement studies have shown that there is correlation in spectrum occupancy across time and frequency [28,29,30]. Therefore, in this work, we consider a CRN utilizing multiple time-slotted primary channels in which the CUs are powered by finite capacity batteries and energy harvesters. Because of constraints in hardware, the CUs under consideration cannot sense and transmit on more than one channel at the same time. Further, practical factors, such as frequency-switching delay, energy-switching cost, correlation in spectrum occupancy across time and frequency and errors in spectrum sensing, are also considered. For a practical scenario where the arrival of energy packets and battery capacity are finite, we propose a scheme to find the optimal sensing schedule, consisting of (i) deciding on the action (*i.e.*, be active or stay silent) and (ii) finding the optimal channel sensing order; and to find the optimal transmission energy corresponding to each channel in the sensing order in order to maximize expected throughput over multiple time slots.

The main contributions of this paper are summarized as follows:

- For the addressed scenario, we firstly analyze the expected throughput over multiple time slots to find the recursive expressions for maximizing the expected throughput of the CRN. These expressions allow the CUs to decide the optimal action, the optimal channel sensing order and the optimal transmission energy set corresponding to channels in the sensing order.
- Secondly, we propose an algorithm based on the recursive expressions above. The algorithm is described in detail by the flow chart and pseudocode.
- Finally, we evaluate the effectiveness of the proposed scheme via simulation by comparing it with that of reference schemes in the literature.

The rest of the paper is organized as follows. The system description is given in Section 2. Throughput analysis and the algorithm are presented in Section 3. Simulation results and the discussion are given in Section 4. The paper is concluded in Section 5.

## 2. System Description

**Figure 1:**
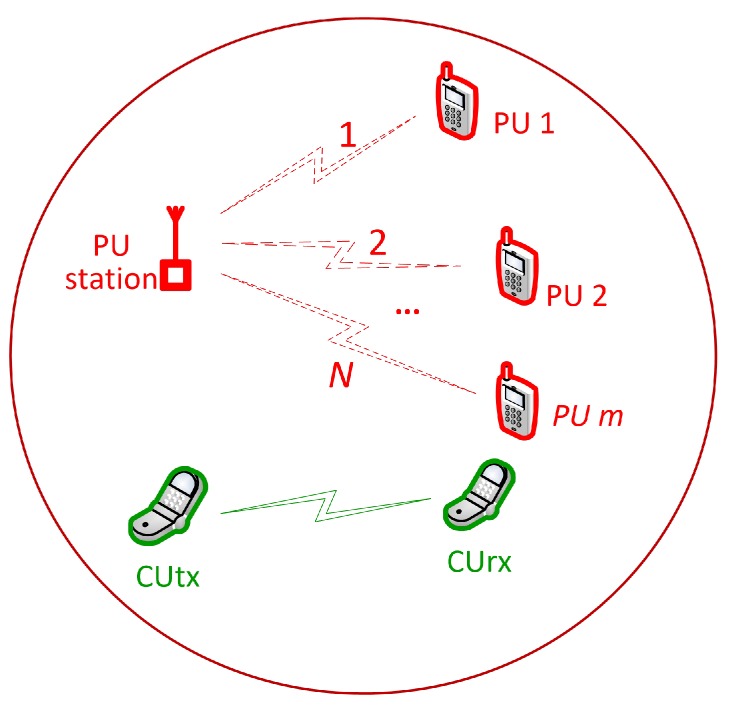
System model.

We consider an energy-harvesting powered CRN consisting of a pair of cognitive users (CUs) utilizing *N* time-slotted primary channels, as shown in Figure 1. These primary channels are assumed to be correlated in spectrum occupancy across time and frequency [7,28,29,30]. Thus, the state of *N*-channels can be described as follows. A state of a channel is represented as $\left\{n,X\right\}|X\in \left\{H0,H1\right\}$, where *n* is the channel index and H0 and H1 correspond to the hypotheses that the PU’s activity is absent or present, respectively. Let $Q=\left\{Q_{0},Q_{1},\ldots ,Q_{M-1}\right\}$, where $M=2^{N}$, be the vector of all possible states of *N* channels. Each element, $Q_{j}$, where j=0,1,2,...,M-1, represents a state of *N* channels. For example, Table 1 shows state *Q* for *N* channels when N=2.

**Figure 2:**
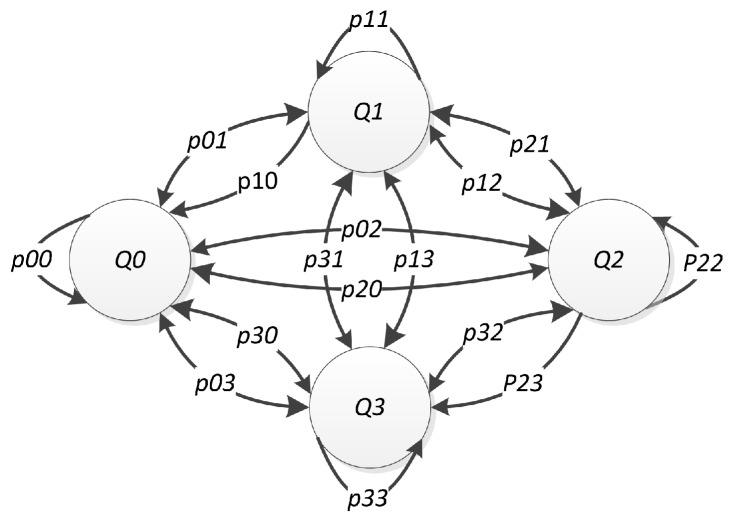
The transition model for the state of *N* channels between two time slots when N=2.

The transition in the state of *N* channels between two adjacent time slots is modeled by a discrete-time Markov chain, as shown in Figure 2 when N=2. Matrix $P_{MxM}\left(p_{ij}\right)$ is defined as the transition matrix, where each element $p_{ij}|i,j=0,1,2,...,M-1$ shows the transition probability from state $Q_{i}$ (the state in the current time slot) to state $Q_{j}$ (the state in the next time slot) [28].

In practical systems, a CU cannot tune its radio from one channel to another immediately. Thus, both channel-switching delay and channel-switching energy consumption (switching cost) are considered. Let $e_{CS}$ and $\tau_{CS}$ be the switching cost and the normalized channel-switching delay (channel-switching delay is normalized by the duration of a time slot) for each step (a predefined frequency distance from the current channel to the destination channel), respectively. The duration of $\tau_{CS}$ is assumed to be much less than the duration of a time slot. Without loss of generality, it can be assumed that all *N* channels are contiguous, and a step is a frequency gap from a current channel to its adjacent channel.

In this paper, we consider a scenario in which each CU is powered by an energy harvester. During a time slot, harvested energy is stored in a rechargeable battery with a finite capacity, $e^{Bat}$ (energy packets), that can be used starting from the next time slot. At time slot *t*, harvested energy $e^{hv}\left(t\right)$ takes values from a finite number of energy packets, as follows:

(1) $$e^{hv}\left(t\right)=e_{i}^{hv}\in \left\{e_{1}^{hv},e_{2}^{hv},...,e_{\zeta }^{hv}\right\}$$

where $0\leq e_{1}^{hv}<e_{2}^{hv}<...<e_{\zeta }^{hv}\leq e^{Bat}$.

The probability mass function of $e^{hv}\left(t\right)$ is given as follows:

(2) $$p^{hv}\left(k\right)=\mathrm{Pr}\left[e^{hv}\left(t\right)=e_{k}^{hv}\right],k=1,2,...,\zeta$$

In addition, the CU cannot sense or transmit more than one channel simultaneously because of the constraints in hardware. Thus, in order to use the energy efficiently under the limit of the arrival of harvested energy packets and the battery capacity of the CU, it is important for the CU to find the optimal channel sensing order and subsequently to decide the optimal transmission energy if a free channel is found.

In the proposed scheme, the operation of the CU in each time slot can be described as follows. First of all, if the remaining energy in the battery is not enough for sensing a channel, the CU has to stay silent during the remainder of the current time slot. Secondly, if the remaining energy in the battery is sufficient, the CU will find the optimal action for maximizing the expected throughput for the next *K* time slots. The action is defined as either the silent state (Silent) or the active state (Active). Silent means that the CU will stay silent during the remainder of the current time slot, whereas Active means that the CU will sense the primary channels one by one to find a free channel for transmission. For the active state, the CU also finds the optimal sensing order of channels and the optimal transmission energy set corresponding to the channels in sensing order. After the optimal action, the optimal sensing order and the optimal transmission energy set are obtained; if the action is Silent, the CU will stay silent during the remainder of the current time slot to save energy for use in the next time slots; otherwise, the CU will follow the sensing order to sense channels one by one to find a free channel for data transmission. The sensing process ends when one of the following happens: (i) a free channel is found, and the CU utilizes the remaining duration of the current time slot for its data transmission with the optimal transmission energy decided upon in the second step; (ii) the current channel is sensed as busy, but the remaining energy is not enough for the CU to sense another channel; (iii) all of the primary channels are sensed, but no channel is found free. At the end of each time slot, based on the consumed energy and harvested energy in the current time slot, the CU will update the remaining energy in the battery for use in the next time slots. The target of this work is to maximize the average throughput of the CRN during its entire operating duration.

**Figure 3:**
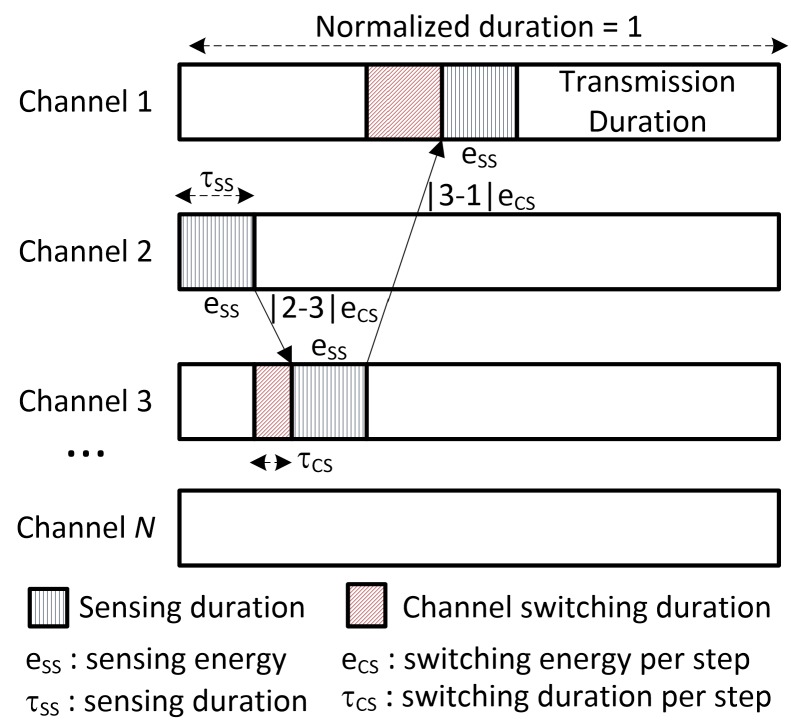
Frame structure of a time slot.

The frame structure of a time slot and an example of the CU’s operation in the time slot are shown in Figure 3. After the channel sensing order is obtained, the CU follows the order to perform the sensing process. In this example, the CU starts sensing at Channel 2 and finds that Channel 2 is busy. Thus, the CU switches to Channel 3 and finds that this channel is not vacant. Therefore, the CU continues to switch and sense another channel. In this case, Channel 1 is found free on the third attempt, and thereby, the CU utilizes the remaining duration of the current time slot for its transmission. Obviously, if the CU finds a free channel in fewer sensing attempts, the transmission interval for the processed time slot will be longer; hence, this channel is utilized more efficiently, and the throughput is improved. In addition, the CU consumed less energy for spectrum sensing and spectrum switching, so it saves more energy for the data transmission process, and the throughput is improved, as well. On the other hand, (i) if the number of sensed channels in the current time slot is fewer, the CU will have less information about the states of all channels in order to update their states in the next time slot, which would benefit the decisions in the next time slot, and (ii) since the battery capacity and the arrival of harvested energy packets are finite, if the CU consumes all of the remaining energy for its data transmission to maximize throughput in the current time slot, it may lack energy for use in the next time slots. Therefore, overall throughput will not be improved, since the active duration of the CU is, practically speaking, much longer than one time slot.

This paper considers the long-term operation of the CU. According to the above analysis, considering the expected throughput over the next *K* time slots, we are interested in finding (i) the optimal channel sensing schedule (consisting of finding the optimal action and sensing order of channels) and (ii) the optimal transmission energy set corresponding to the channels in the sensing order for the operation of the CU. The target of the proposed scheme is to maximize overall throughput for the entire operation time of the CU. The formulas of the proposed scheme are investigated in the next section.

## 3. Throughput Analysis and Algorithm

In this section, the expected throughput of the CRN over the next *K* time slots is analyzed and formulated. Based on the formulated expressions, we propose an algorithm to find the optimal action, the optimal sensing order of channels and its corresponding optimal transmission energy set in order to maximize the expected throughput over the next *K* time slots.

### 3.1. Throughput Analysis

In practical CRNs, false alarms and misdetection events are always inevitable when the CU performs spectrum sensing on a channel. A false alarm reduces spectrum utilization, whereas a misdetection event may cause a collision between the transmission of the CU and that of the PU. Appropriate sensing schemes and spectrum sensing parameters are required to maximize the throughput of the CRN under the collision constraint on primary channels. To obtain a sensing scheme with low complexity, the probability of detection is fixed according to the collision constraint on the primary channel. The probability of a false alarm when the CU senses channel *n* is calculated as follows [6]:

(3) $$p_{f}\left(n\right)=Q\left(\sqrt{2SNR(n)+1}Q^{-1}\left(p_{d}\left(n\right)\right)+\sqrt{\tau_{SS}f_{s}}SNR\left(n\right)\right)$$

where $f_{s}$ denotes the sampling frequency of the sensing device and SNR(n) denotes the channel gain from the PU transmitter to the sensing device.

Let $\mu =\left\{\mu_{0},\mu_{1},\ldots ,\mu_{M-1}\right\}$, where $M=2^{N}$, be the belief vector, which stands for the state probabilities corresponding to the states of *N* channels, $Q=\left\{Q_{0},Q_{1},\ldots ,Q_{M-1}\right\}$. Each element $\mu_{j}$ represents the probability that the state of *N* channels is $Q_{j}$. The belief vector θ(n) is used to calculate the probability that channel *n* is available as follows:

(4) $$\theta \left(n\right)=\frac{{\left.\sum_{j=0}^{M-1}\mu_{j}\right|}_{\left\{n,H0\right\}\in Q_{j}}}{\sum_{j=0}^{M-1}\mu_{j}}$$

Vector ***μ*** needs to be updated after each sensing or whenever the CU proceeds to the next time slot. When channel *n* is sensed, each element of vector ***μ*** is updated according to Equations (5) and (6) as follows:

μj=Pn,H0PQj\n,H0(5)=Pn,H0PQj\n,H0θ0nθ0n=θnμj0θ0n,ifn,H0∈Qj

μj=Pn,H1PQj\n,H1(6)=Pn,H1PQj\n,H11-θ0n1-θ0n=1-θnμj01-θ0n,ifn,H1∈Qj

where j=0,1,2,...,M-1, $\mu_{j}$${}^{0}$ denotes the belief before being updated and *θ*${}^{0}$$\left(n\right)$ denotes the value of $\theta \left(n\right)$ before sensing. $\theta \left(n\right)$ can be calculated according to the sensing outcome on channel *n* and the feedback after transmission on channel *n* as follows:

When channel *n* is sensed and found busy, $\theta \left(n\right)$ can be calculated as:

(7) $$\theta \left(n\right)=\frac{\theta^{0}\left(n\right)p_{f}\left(n\right)}{\theta^{0}\left(n\right)p_{f}\left(n\right)+\left(1-\theta^{0}\left(n\right)\right)p_{d}\left(n\right)}.$$

When channel *n* is sensed and found free, but the CU does not transmit because of low remaining energy, $\theta \left(n\right)$ can be calculate as:

(8) $$\theta \left(n\right)=\frac{\theta^{0}\left(n\right)\left(1-p_{f}\left(n\right)\right)}{\theta^{0}\left(n\right)\left(1-p_{f}\left(n\right)\right)+\left(1-\theta^{0}\left(n\right)\right)\left(1-p_{d}\left(n\right)\right)}.$$

When channel *n* is sensed and found free and the CU transmits its data on channel *n*, $\theta \left(n\right)$ should be one if the transmission is successful and zero, otherwise.

When the CU finishes processing on time slot *t* and proceeds to the next time slot, t+1, each element of vector ***μ*** is updated according to Equation (9) as follows based on the Markov chain transition matrix.

(9) $$\mu_{j}\left(t+1\right)=\sum_{i=0}^{M-1}\mu_{i}\left(t\right)p_{ij}$$

Let $S\subseteq \left\{1,2,\ldots ,N\right\}$ be the set of sensed channels that are **arranged in sensed order** from the beginning of a slot. When the CU senses a channel, it will append that channel to set S. Suppose that the CU’s radio hardware is on channel $n_{0}$ at the beginning of the current time slot. The information in $n_{0}$ and S is used to calculate the interval and cost for channel switching, and the interval and cost for spectrum sensing in the current time slot.

At time slot *t*, when channel *n* is sensed as free, the normalized transmission rate (nat/Hz) (also called throughput) on channel *n* is given as follows:

(10) $$R^{t}\left(n,n_{0},S,e^{total}\right)=\left(1-\sum \tau_{SS}-\sum \tau_{CS}\right)\ln\left(1+\frac{e_{opt}^{tr}\left(n\right)}{\left(1-\sum \tau_{SS}-\sum \tau_{CS}\right)}\gamma \left(n\right)\right)$$

where $e^{total}$ denotes the total number of energy packets in the battery that the CU can spend for the current time slot; $\sum \tau_{SS}$ and $\sum \tau_{CS}$ denote the total sensing duration and the total switching duration from the beginning of the current time slot, respectively, until channel *n* is found free; γ(n) is defined as the gain of channel *n* between the CU transmitter and the CU receiver when transmitted energy is unity; and $e_{opt}^{tr}\left(n\right)$ denotes the optimal transmission energy for channel *n* at the time channel *n* was found free. The value of $e_{opt}^{tr}\left(n\right)$ is decided based on the remaining energy packets in the battery at the time channel *n* is found free.

For a single time slot, the expected throughput when a CU starts to sense channel *n* is given as follows:

E1n(n0,S,μ,etotal)=θ(n)1-pf(n)1-∑τSS-∑τCSln1+eopttr(n)1-∑τSS-∑τCSγ(n)︷(1)(11)+θ(n)pf(n)+1-θ(n)pd(n)E1(n0,S∪n,μb,etotal)︸(2)

where $\mu^{b}$ denotes the updated value of vector ***μ***, according to Equations (5) and (6), when channel *n* is sensed as busy. Equation (11) consists of two parts: Part (1) is the expected throughput when channel *n* is found free and the transmission on channel *n* is successful (via the feedback from the CU receiver to the CU transmitter); Part (2) is the expected throughput when channel *n* is sensed as busy. Note that when channel *n* is found free, but the transmission on this channel is unsuccessful (the CU transmitter receives a negative acknowledgment from CU receiver or does not receive feedback), the expected throughput is zero; thus, it is not shown in Equation (11). To calculate $e_{opt}^{tr}\left(n\right)$, let $e^{tr}\left(n\right)$ be the amount of transmission energy that the CU uses on channel *n*. We assume that $e^{tr}\left(n\right)$ takes values from a finite number of energy packets as follows:

(12) $$e^{tr}\left(n\right)=e_{i}^{tr}\in \left\{e_{0}^{tr}=0,e_{1}^{tr},e_{2}^{tr},...,e_{\psi -1}^{tr}\right\}.$$

Based on the remaining energy in the battery when channel *n* is found free, $e_{opt}^{tr}\left(n\right)$ can be given as follows:

(13) $$e_{opt}^{tr}\left(n\right)=e_{i^{*}}^{tr}\left(n\right)=\underset{i\in \left\{0,1,2,...,\psi -1\right\},e_{i}^{tr}\leq \left(e^{total}-\sum e_{SS}-\sum e_{CS}\right)}{arg\;\max}\left(e_{i}^{tr}\right)$$

where $\sum e_{SS}$ and $\sum e_{CS}$ denote the energy packets consumed for spectrum sensing and channel switching from the beginning of the current time slot to the time when channel *n* is found free, respectively. $\sum e_{SS}$ and $\sum e_{CS}$ can be calculated based on the information in *n*, $n_{0}$ and S.

The expected throughput when CU starts to sense from every channel $n\in \left\{\left\{1,2,...,N\right\}\backslash S\right\}$ can be calculated by using Equation (11), and the optimal starting channel for sensing can be obtained with Equation (14) as follows:

(14) $$E_{1}^{n*}\left(n_{0},S,\mu ,e^{total}\right)=\underset{n\in \left\{\left\{1,2,...,N\right\}\backslash S\right\}}{arg\;\max}\left(E_{1}^{n}\left(n_{0},S,\mu ,e^{total}\right)\right)$$

We note that, to calculate $E_{1}^{n}\left(n_{0},S,\mu ,e^{total}\right)$ in Equation (11), we need to calculate $E_{1}\left(n_{0},S\cup \left\{n\right\},\mu_{b},e^{total}\right)$, which is a sub-optimal problem when the CU senses channel *n* and finds it busy. To obtain $E_{1}\left(n_{0},S\cup \left\{n\right\},\mu_{b},e^{total}\right)$, we need to recursively calculate according to Equations (11)–(14). The loop terminates when we reach the last channel of the current time slot, when all channels have already been sensed and $E_{1}\left(n_{0},S\cup \left\{n\right\},\mu_{b},e^{total}\right)$ is zero.

Since the active duration of a CU in the CRN is in practice much longer than one time slot, this work considers the throughput for long-term operation of the CU. We focus on finding the optimal action (Active or Silent), the sensing order of channels and the optimal transmission energy set corresponding to the channels in the sensing order for the operation of the CU to maximize overall throughput over the next *K* time slots. The formulation analysis and an algorithm for the addressed problem are described as follows.

Let $E_{K}\left(n_{0},S,\mu_{K},e_{K}^{total}\right)$ be the expected throughput for the next *K* time slots, and let $E_{K}^{n}\left(n_{0},S,\mu_{K},e_{K}^{total}\right)$ be the expected throughput for the next *K* time slots when the CU starts to sense channel *n*. At any one time, the CU may decide to be either in the silent state to save energy for use in the next time slots or in the active state to sense a channel and subsequently transmit if the channel is found free. The decision is based on the remaining energy in the battery and the expected throughput for the two states. Let $Ea_{K}^{n}\left(n_{0},S,\mu_{K},e_{K}^{total}\right)$ and $Es_{K}^{n}\left(n_{0},S,\mu_{K},e_{K}^{total}\right)$ be the expected throughput for the next *K* time slots when the CU is in Active and Silent, respectively. $Ea_{K}^{n}\left(n_{0},S,\mu_{K},e_{K}^{total}\right)$ can be calculated by extending Equation (11) as follows:

EaKnn0,S,μK,eKtotal=θ(n)1-pf(n)1-∑τSS-∑τCSln1+eopttr(n)1-∑τSS-∑τCSγ(n)+EK-1(n0=n,S=∅,μK-1f,eK-1total)︷(1)+1-θ(n)1-pd(n)EK-1(n0=n,S=∅,μK-1b,eK-1total)︸(2)+θ(n)pf(n)+1-θ(n)pd(n)EK(n0,S∪n,μKb,eKtotal)︸(3)=PAck1-∑τSS-∑τCSln1+eopttr(n)1-∑τSS-∑τCSγ(n)+EK-1n0=n,S=∅,μK-1f,eK-1total︷(1)(15)+PNackEK-1n0=n,S=∅,μK-1b,eK-1total︸(2)+PBusyEKn0,S∪n,μKb,eKtotal︸(3)

where $\mu^{f}$ and $\mu^{b}$ denote the updated values of vector ***μ***, according to Equations (5) and (6), when channel *n* is sensed free and busy, respectively; $\mu_{K-1}$ denotes the undated values of $\mu_{K}$, according to Equation (9); $P_{Ack}$ and $P_{Nack}$ denote the probabilities that the transmission is successful and unsuccessful, respectively, when channel *n* is sensed as free; and $P_{Busy}$ denotes the probability that channel *n* is sensed as busy. Equation (15) consists of three parts: Part (3) is the expected throughput when channel *n* is sensed as busy. When channel *n* is found free, Parts (1) and (2) are the expected throughput when transmission on channel *n* is successful and unsuccessful, respectively (according to the feedback from the CU receiver to the CU transmitter). Note that Part (2) is the expected throughput when the transmission in the current time slot is unsuccessful and collision with the primary user happened on channel *n*; thus, only the throughput for the next K-1 time slots is achieved. In Equation (15), $e_{K-1}^{total}$ denotes the updated value of $e_{K}^{total}$ after consuming energy for the operation in the current time slot and compensation by the harvested energy at the end of the time slot, which can be calculated as follows:

(16) $$e_{K-1}^{total}=\min\left(e^{Bat},e_{K}^{total}-\left(\sum e_{SS}+\sum e_{CS}+e_{opt}^{tr}\left(n\right)\right)+e_{i}^{hv}\right)$$

where $e_{i}^{hv}\in \left\{e_{1}^{hv},e_{2}^{hv},...,e_{\zeta }^{hv}\right\}$ denotes the energy packets harvested during the current time slot. We can see that the decision about transmission energy packets on channel *n* has an effect on throughput in the next time slots. Therefore, to find $e_{opt}^{tr}\left(n\right)$, we solve a sub-optimal problem by maximizing the summation of Part (1) and Part (2) in Equation (15) as follows. Let $R_{i}\left(n,e_{i}^{tr}\right)$ be $\left(1-\sum \tau_{SS}-\sum \tau_{CS}\right)\ln\left(1+\frac{e_{i}^{tr}\left(n\right)}{\left(1-\sum \tau_{SS}-\sum \tau_{CS}\right)}\gamma \left(n\right)\right)$. The optimal value of $e_{opt}^{tr}\left(n\right)$ can be given as:

(17) $$\begin{array}{c} e_{opt}^{tr}\left(n\right)=e_{i*}^{tr}=\underset{e_{i}^{tr}\in \left\{e_{0}^{tr}=0,e_{1}^{tr},e_{2}^{tr},...,e_{\psi -1}^{tr}\right\}}{arg\;\max}\left(\begin{array}{c} P_{Ack}\left(R_{i}\left(n,e_{i}^{tr}\right)+E_{K-1}\left(n_{0}=n,S=\emptyset ,\mu_{K-1}^{f},e_{K-1}^{total}\right)\right)\\ +P_{Nack}E_{K-1}\left(n_{0}=n,S=\emptyset ,\mu_{K-1}^{b},e_{K-1}^{total}\right) \end{array}\right) \end{array}$$

where $e_{K-1}^{total}$ can be calculated according to Equation (16). In the state Active, the expected throughput $Ea_{K}^{n}\left(n_{0},S,\mu_{K},e_{K}^{total}\right)$ when the CU starts to sense from every channel $n\in \left\{\left\{1,2,...,N\right\}\backslash S\right\}$ can be calculated by using Equations (15)–(17).

When the CU decides to stay in the state Silent during the current time slot, the expected throughput can be obtained with Equation (18). Since the CU stays silent during the current time slot, $n_{0}$ is not changed in the next time slot.

(18) $$Es_{K}\left(n_{0},S,\mu_{K},e_{K}^{total}\right)=E_{K-1}\left(n_{0},S=\emptyset ,\mu_{K-1},e_{K-1}^{total}\right)$$

where $\mu_{K-1}$ denotes the updated value of $\mu_{K}$ for moving to the next time slot, according to Equation (9).

After the expected throughput for the state Active and Silent are obtained, the action for channel *n* can be obtained with Equation (19) as follows:

(19) $$E_{K}^{n}\left(n_{0},S,\mu_{K},e_{K}^{total}\right)=\max\left(Ea_{K}^{n}\left(n_{0},S,\mu_{K},e_{K}^{total}\right),Es_{K}\left(n_{0},S,\mu_{K},e_{K}^{total}\right)\right)$$

The optimal decision and the optimal channel n* for starting the sensing process with its corresponding optimal transmission energy $e_{opt}^{tr}\left(n*\right)$ can be obtained with Equation (20).

(20) $$E_{K}\left(n_{0},S,\mu_{K},e_{K}^{total}\right)=E_{K}^{n*}\left(n_{0},S,\mu_{K},e_{K}^{total}\right)=\underset{n\in \left\{\left\{1,2,...,N\right\}\backslash S\right\}}{argmax}\left(E_{K}^{n}\left(n_{0},S,\mu_{K},e_{K}^{total}\right)\right)$$

We note that, to calculate $Ea_{K}^{n}\left(n_{0},S,\mu_{K},e_{K}^{total}\right)$ in Equation (15) and $Es_{K}\left(n_{0},S,\mu_{K},e_{K}^{total}\right)$ in Equation (18), we need to find $e_{opt}^{tr}\left(n\right)$, $E_{K-1}\left(n_{0}=n,S=\emptyset ,\mu^{f},e_{K-1}^{total}\right)$, $E_{K-1}\left(n_{0}=n,S=\emptyset ,\mu^{b},e_{K-1}^{total}\right)$, $E_{K}\left(n_{0},S\cup \left\{n\right\},\mu^{b},e_{K}^{total}\right)$ and $E_{K-1}\left(n_{0},S=\emptyset ,\mu_{K-1},e_{K-1}^{total}\right)$, which are sub-optimal problems that can be recursively calculated according to Equations (15)–(20). The loop terminates when we reach the last time slot (K=1), where we can solve the problem for a single time slot according to Equations (11)–(14).

### 3.2. The Proposed Algorithm for Maximizing the Expected Throughput

Up to now, we have formulated the optimal operation of the CU to maximize the expected throughput of the CRN over the next *K* time slots. First of all, we calculate the expected throughput and the corresponding optimal transmission energy and find the action, *i.e.*, the state Active or Silent for each separate channel according to Equations (15)–(19). After that, the optimal action and the optimal channel n* for starting the sensing process with its corresponding transmission energy $e_{opt}^{tr}\left(n*\right)$ are obtained based on Equation (20). Note that if the remaining energy on the battery is not enough for sensing such a channel, the CU must stay in the state Silent during the current time slot without calculating the expected throughput.

**Figure 4:**
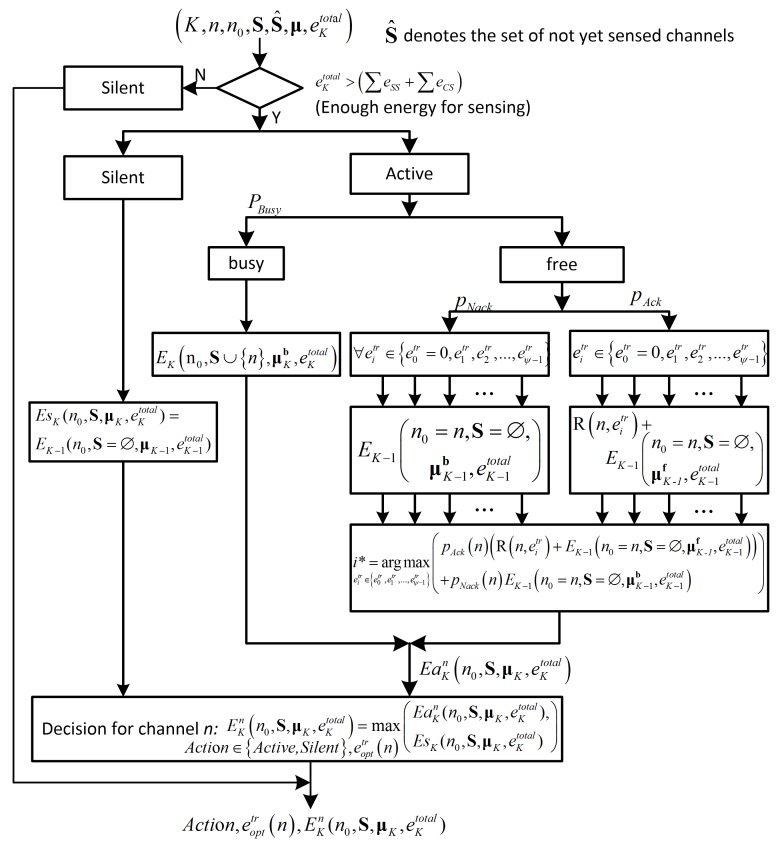
The flow chart of the proposed algorithm.

In this section, we propose an algorithm based on Equations (15)–(20) to find the expected throughput $E_{K}^{n}\left(n_{0},S,\mu_{K},e_{K}^{total}\right)$ with its corresponding optimal transmission energy $e_{opt}^{tr}\left(n\right)$ when the CU starts its sensing-process on channel *n*. In addition, the optimal action (*i.e.*, Active or Silent) for channel *n* is also decided based on this algorithm. The pseudocode of the proposed algorithm is given in the Algorithm 1, and the flow chart is shown in Figure 4. For simplicity, the flow chart shows the processing for one channel *n*. After that, based on the results for each separate channel, we obtain the optimal action and the optimal staring channel n* with its corresponding transmission energy $e_{opt}^{tr}\left(n*\right)$ according to Equation (20). The optimal sensing order of all channels and the optimal transmission energy set corresponding to the order of the channels can be obtained based on the recorded optimal transitions whenever we make a decision based on Equation (20).
**Algorithm 1:** The pseudocode of the proposed algorithm for finding the expected throughput $E_{K}^{n}\left(n_{0},S,\mu ,e_{K}^{total}\right)$, the optimal transmission energy $e_{opt}^{tr}\left(n\right)$ and the action for channel *n*.**Notations**:*K* denotes the number of slots for calculating the channel sensing order.$S\subseteq \left\{1,2,\ldots ,N\right\}$ denotes the set of sensed channels, which are arranged in sensed order.$\hat{S}$ denotes the set of not yet sensed channels.$e_{K}^{total}$ denotes the packets of energy in the battery at the beginning of each time slot.$e_{SS}$ and $e_{CS}$ denote the energy packets consuming for sensing per channel and channel switching per step, respectively.**Input**: $K,n,n_{0},S,\mu_{K},e_{K}^{total}$; **Output**: Action (Active or Silent), $E_{K}^{n}\left(n_{0},S,\mu_{K},e_{K}^{total}\right)$ and $e_{opt}^{tr}\left(n\right)$.1: $S=S\cup \left\{n\right\}$; $\hat{S}=\left\{1,2,\ldots ,N\right\}\backslash S$; use Equation (4) to calculate θ(.) for each channel.2: **if**
$e_{K}^{total}<\left(\sum e_{SS}+\sum e_{CS}\right)$ // Remaining energy is not enough for sensing channel *n*3:  Action = Silent, $E_{K}^{n}\left(n_{0},S,\mu_{K},e_{K}^{total}\right)=0$, $e_{opt}^{tr}\left(n\right)=0$4: **else**// Calculate the expected throughput when Action = Silent5:  **if**
K=1 // the current time slot is the last time slot6:   $Es_{K}\left(n_{0},S,\mu_{K},e_{K}^{total}\right)=0$7:  **else**8:   Use Equation (9) to update $\mu_{K-1}$; use Equation (16) to update $e_{K-1}^{total}$; and use Equation (18) to calculate $Es_{K}\left(n_{0},S,\mu ,e_{K}^{total}\right)$= $E_{K-1}\left(n_{0},S=\emptyset ,\mu_{K-1},e_{K-1}^{total}\right)$, recursively.9:  **end if**// Calculate the expected throughput when the CU stays in the state Active10:  **if**
K=1 // the current time slot is the last.11:   $E_{K-1}\left(n_{0}=n,S=\emptyset ,\mu_{K-1}^{f},e_{K-1}^{total}\right)=0$, $E_{K-1}\left(n_{0}=n,S=\emptyset ,\mu_{K-1}^{b},e_{K-1}^{total}\right)=0$.12:   **if**
$\left(\hat{S}=\emptyset \right)$ .13:    set $E_{K}\left(n_{0},S\cup \left\{n\right\},\mu_{K}^{b},e_{K}^{total}\right)=0$, use Equation (13) to calculate $e_{opt}^{tr}\left(n\right)$, and use Equation (15) to calculate $Ea_{K}^{n}\left(n_{0},S,\mu_{K},e_{K}^{total}\right)$.14:   **else**15:    calculate $E_{K}\left(n_{0},S\cup \left\{n\right\},\mu_{K}^{b},e_{K}^{total}\right)$ recursively, use Equation (13) to calculate $e_{opt}^{tr}\left(n\right)$ and use Equation (15) to calculate $Ea_{K}^{n}\left(n_{0},S,\mu_{K},e_{K}^{total}\right)$.16:   **end if**17:  **else**18:   calculate $E_{K-1}\left(n_{0}=n,S=\emptyset ,\mu_{K-1}^{f},e_{K-1}^{total}\right)$ and $E_{K-1}\left(n_{0}=n,S=\emptyset ,\mu_{K-1}^{b},e_{K-1}^{total}\right)$, recursively (use Equation (16) to update $e_{K-1}^{total}$); then combine with Equation (17) to calculate $e_{opt}^{tr}\left(n\right)$19:   **if**
$\left(\hat{S}=\emptyset \right)$ set $E_{K}(n_{0},S\cup \left\{n\right\},\mu_{K}^{b},e_{K}^{total}=0$20:   **else** calculate $E_{K}\left(n_{0},S\cup \left\{n\right\},\mu_{K}^{b},e_{K}^{total}\right)$ recursively21:   **end if**22:   use Equation (19) to decide the Action (Active or Silent), and calculate $E_{K}^{n}\left(n_{0},S,\mu_{K},e_{K}^{total}\right)$ and $e_{opt}^{tr}\left(n\right)$23: **end if**

To calculate the complexity of the proposed algorithm, first of all, we analyze the computational complexity for a single time slot as follows. When the CU processes a channel, there are three possible cases. Firstly, (i) the CU stays silent during the current time slot. In this case, no calculation is needed. Secondly, (ii) if the CU stays active to sense the channel and finds it busy, it will process another channel. Lastly, (iii) if the channel is sensed and found free, the CU starts its transmission process. In Case (iii), there are *ψ* possible cases of transmission levels of energy. Based on the above analysis, when the CU processes a channel, there are *ψ* possible cases of transmission and one possible case that the CU will process another channel. Therefore, the computational complexity that the CU processes *N* channels will be given as $O\left(\left(\psi +1\right)\left(N+\left(N-1\right)+\left(N-2\right)+...+1\right)\right)=O\left(\psi N\right)$. Finally, for the next *K* time slots, the total computational complexity for which the CU processes *N* channels can be calculated as $O\left({\left(\psi N\right)}^{K}\right)$.

## 4. Simulation Results and Discussion

In this section, we demonstrate the effectiveness of the proposed scheme via simulation. The simulation parameters are summarized in Table 2. Since we failed to find existing schemes that are similar to the proposed scheme, the performance of the proposed scheme is compared to those of two schemes described as follows.

- **Random scheme**: In this scheme, the channel is selected randomly for sensing.
- **Reference scheme**: This scheme is the combination of the two schemes proposed in [11,26].

At the beginning of each time slot, the channel sensing order is decided to maximize the throughput of the CRN. In this scheme, the correlation in spectrum occupancy across time and frequency was considered. However, harvesting energy, limited capacity of the battery, channel switching delay and switching cost were not considered.

In addition, the random and reference schemes will operate as follows when the harvesting energy and limited capacity of battery are considered. The CU only stays active and senses a channel if the remaining energy in the battery is sufficient. Otherwise, it stays silent during the remaining of the current time slot. Whenever a channel is sensed as free, based on the remaining energy in the battery, the CU will spend as much energy as possible for transmission to maximize throughput for the current time slot. At the end of each time slot, the remaining energy in the battery and channel states are updated the same as in our proposed scheme. The updated information will be utilized in the next time slot. It is noteworthy that the decision of channel sensing order of the reference scheme is nearly similar to the proposed scheme when K=1.

**Figure 5:**
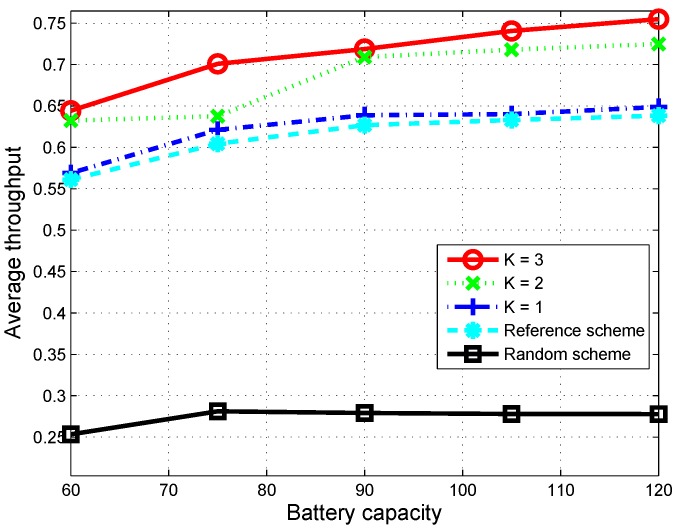
Average throughput according to the battery capacity over 1000 time slots when the simulation is run 50 times.

Figure 5 shows the average throughput of the proposed scheme with different values of *K* according to the battery capacity of the CU. We can see that the throughput of the proposed scheme is greatly improved in comparison to those of the random scheme and the reference scheme. That is, the proposed scheme can provide 15% to 20% more throughput improvement than the reference scheme when K=3. When the battery capacity increases, the average throughput of the proposed scheme is improved significantly, whereas that of the random scheme is not improved. This can be explained as follows. For the random scheme, when a channel is sensed as free, the CU spends the maximum allowable energy for its transmission to maximize throughput in the current time slot. Since the arrival of harvested energy packets is limited, the CU may stay silent, due to the lack of energy for use in the next time slots. On the other hand, the proposed scheme considers the optimal transmission energy spent in the current time slot, sparing energy for use in the next time slot. Therefore, the CU in the proposed scheme utilizes the primary channels more efficiently, and the average throughput is improved. In particular, throughput is greatly improved when *K* increases. In is noteworthy that the proposed scheme can provide a small throughput improvement compared to that of the reference scheme, even when K=1, which is mainly due to the fact that the channel switching delay and switching cost are considered in the proposed scheme.

**Figure 6:**
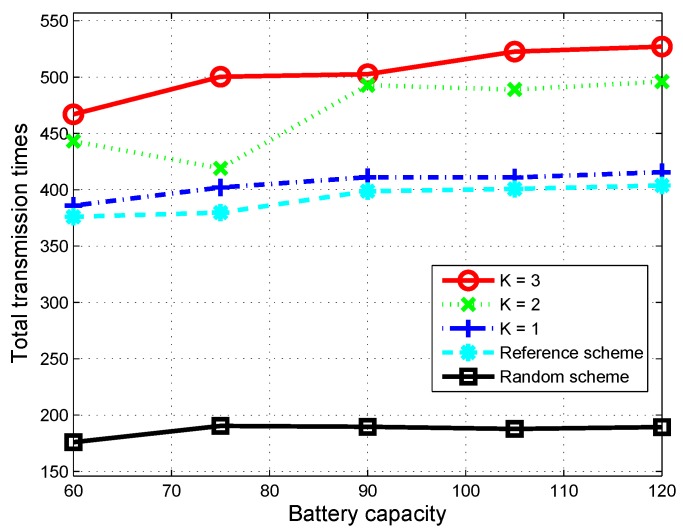
Total transmission times over 1000 time slots according to battery capacity.

For a deeper insight into why the proposed scheme can achieve better performance than the other schemes, we observed total time slots where the CU is in the state Active and transmits its data over 1000 time slots for the random scheme, the reference scheme and the proposed scheme (when *K* is fixed at 1, 2 and 3). The results are shown in Figure 6. We can see that battery capacity has nearly no effect on the random scheme, since the arrival of harvested energy packets in a time slot is limited and may be not enough for the CU to stay active in the next time slots. On the other hand, the proposed scheme considers the limitation on the arrival of harvested energy packets, the limitation on battery capacity and the optimal usage of energy for the next *K* time slots. Therefore, the total number of time slots utilized by the CU for data transmission greatly increase, especially with a high value of *K*. When K=3, for example, total transmission times of the proposed scheme are improved 30% to 35% that of the reference scheme. We can see in the figure that the total transmission times of the proposed scheme increase when *K* increases.

**Figure 7:**
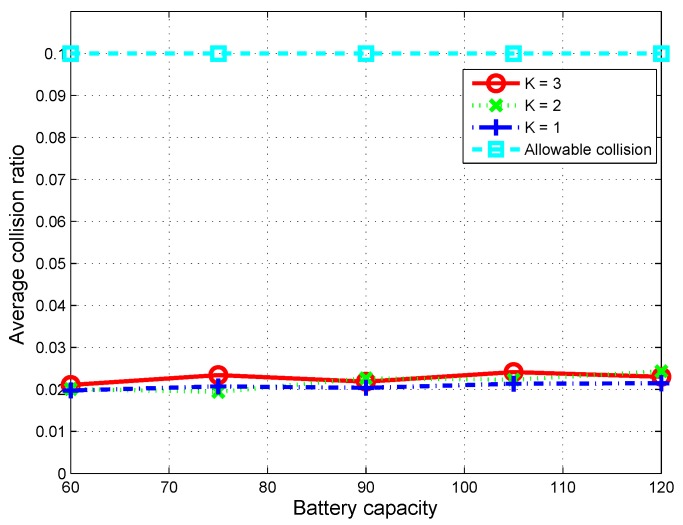
Average collision ratio on a primary channel over 1000 time slots according to battery capacity.

To examine the collision with the PUs’ activities on primary channels, we observed the average collision ratio, which is defined as the total number of collisions over the total number of times the primary channels are not vacant. The simulation results are shown in Figure 7. For a given allowable level of collision, the average collision ratio per channel of the proposed scheme always remains below 25% of the allowable level of 0.1, which means that communications of the PUs can be guaranteed.

**Figure 8:**
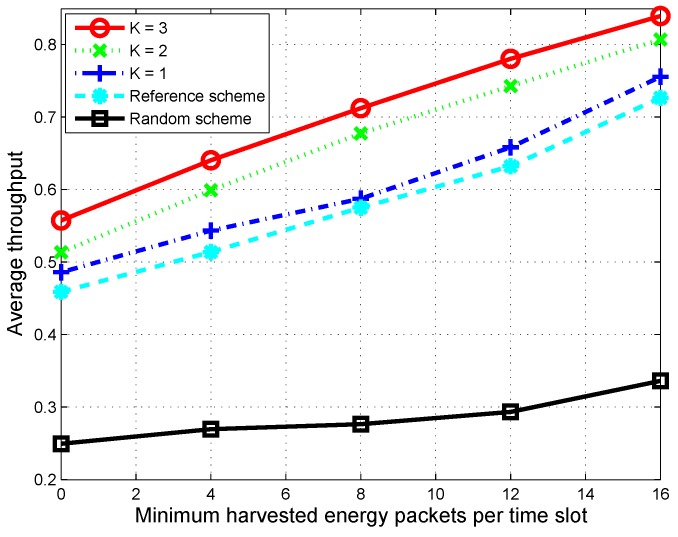
Average throughput over 1000 time slots according to the minimum harvested energy packets per time slot when the maximum harvested energy per time slot is fixed at 40 packets.

In Figure 8, we increase the minimum harvested energy packets per time slot and observe its effect on average throughput when the battery capacity is fixed at 100 packets of energy. For all schemes, since the amount of harvested energy within each time slot increases, the total number of time slots in which the CU can stay active and transmit data increases. Therefore, average throughput is improved according to the increase in the arrival of harvested energy. However, the figure shows that for the proposed scheme, the increasing slopes of average throughput are much faster in comparison to that of the random scheme. The performance of the proposed scheme when K=1 is also better than that of the reference scheme, since the proposed scheme considers the channel switching delay and switching cost when deciding channel sensing order. For the proposed scheme, we can see that throughput greatly improves when *K* increases.

**Figure 9:**
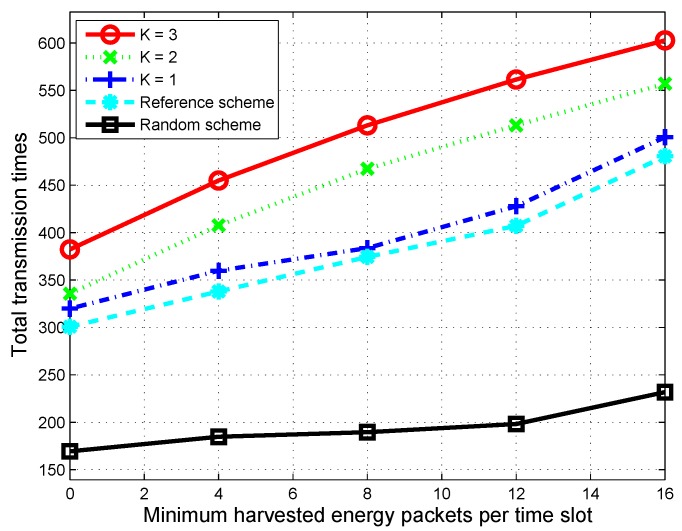
Average throughput over 1000 time slots according to minimum harvested energy packets per time slot when the maximum harvested energy per time slot is fixed at 40 packets.

Figure 9 shows the total number of time slots (over 1000 time slots) where the CU is active and transmits data. We can see that, when more harvested energy packets arrive, the CUs have a greater energy budget to be in the active state; hence, total transmission times increases for all schemes. However, the proposed scheme plans the optimal energy usage for the next *K* time slots; therefore, when the arrival of harvested energy packets increases, the total number of time slots in which the CU stays active significantly increases with a high value of *K*.

**Figure 10:**
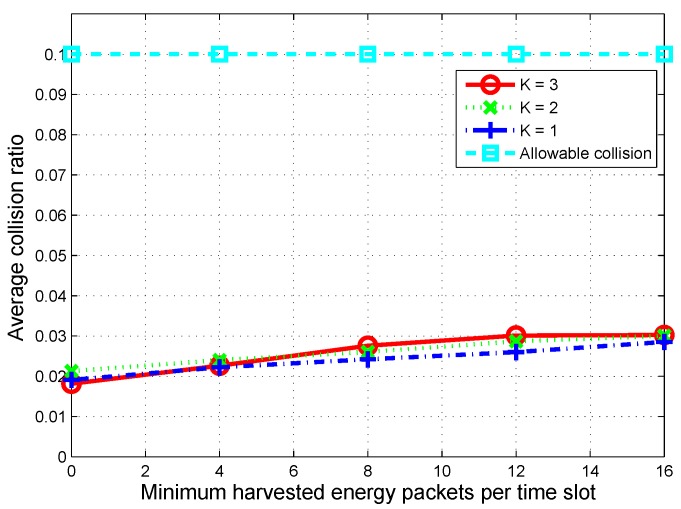
Average collision ratio on a primary channel over 1000 time slots according to the minimum harvested energy packets per time slot when maximum harvested energy per time slot is fixed at 40 packets.

Finally, we observed the average collision ratio according to the minimum harvested energy packets per time slot. The results are shown in Figure 10. We can see that when the arrival of harvested energy increases, the CU has more of a chance to be active. Therefore, the average collision ratio on primary channels increases. However, for a given allowable level of collision, the average collision ratio per channel of the proposed scheme always remains below 30% of the allowable level of 0.1, which means that the communications of the PUs can be guaranteed.

## 5. Conclusions

In the paper, we consider harvesting energy-powered CRNs in which a pair of CUs utilizes multiple time-slotted primary channels. The CUs under consideration cannot sense and transmit on more than one channel at the a time due to hardware constraints in radio devices. For the given scenario, we propose a scheme to find the optimal sensing schedule (*i.e.*, silent state, active state and the optimal sensing order of channels) for the CU, as well as to find the optimal transmission energy set corresponding to the channels in order to maximize the expected throughput over multiple time slots. In addition, practical factors, such as frequency-switching delay, switching cost, the correlation in spectrum occupancy across time and frequency and errors in spectrum sensing, are all considered in the proposed scheme. The simulation results show that throughput of the proposed scheme greatly improves in comparison to that of the random scheme and the reference scheme, especially when K>1. Moreover, the collision ratio on the primary channels is also investigated. As a result, the average collision ratio of the proposed scheme always remains below 30% of the allowable level, which means that the communications of the PUs can be guaranteed.

## Figures and Tables

**Table 1:** The state *Q* of *N* channels when N=2.

State No.	Notations	Elements
0	$Q_{0}$	$\left\{\left\{1,H0\right\},\left\{2,H0\right\}\right\}$
1	$Q_{1}$	$\left\{\left\{1,H0\right\},\left\{2,H1\right\}\right\}$
2	$Q_{2}$	$\left\{\left\{1,H1\right\},\left\{2,H0\right\}\right\}$
3	$Q_{3}$	$\left\{\left\{1,H1\right\},\left\{2,H1\right\}\right\}$

**Table 2:** Simulation parameters.

Symbol	Description	Value
*N*	Number of channels	3
$e^{hv}$	Harvested energy packets per time slot	[040]
$p^{hv}$	Probability mass function of $e^{hv}$	[0.50.5]
$e^{Bat}$	Battery capacity (packets)	80
$e^{tr}$	Transmission energy packets	{0,35,45,55,65}
$\tau_{SS}$	Normalized sensing duration per channel	0.05
$\tau_{CS}$	Normalized switching delay per step	0.01
$e_{SS}$	Sensing cost per channel (packets)	10
$e_{CS}$	Switching cost per step (packets)	2
$p_{d}$	Probability of detection	0.9
$p_{ij}$	Transition probability from state *i* to state *j*	0.03 if i≠j, 0.79 otherwise
